# Neural activation during natural speech and rests in patients with schizophrenia and schizophrenia spectrum disorders—an fMRI pilot trial

**DOI:** 10.3389/fpsyt.2024.1402818

**Published:** 2024-06-13

**Authors:** Wiebke Hahn, Panagiota-Eleni Tsalouchidou, Arne Nagels, Benjamin Straube

**Affiliations:** ^1^ Department of Neurology, Philipps-University Marburg, Marburg, Germany; ^2^ Department of English and Linguistics, Johannes Gutenberg-University Mainz, Mainz, Germany; ^3^ Department of Psychiatry and Psychotherapy, Philipps-University Marburg, Marburg, Germany

**Keywords:** fMRI, spontaneous speech, rest, schizophrenia, psychopathology

## Abstract

**Background:**

In schizophrenia patients, spontaneous speech production has been hypothesized as correlating with right hemispheric activation, including the inferior frontal and superior temporal gyri as speech-relevant areas. However, robust evidence for this association is still missing. The aim of the present fMRI study is to examine BOLD signal changes during natural, fluent speech production in patients with schizophrenia in the chronic phase of their disease.

**Methods:**

Using a case–control design, the study included 15 right-handed patients with schizophrenia spectrum disorders as well as 15 healthy controls. The participants described eight pictures from the Thematic Apperception Test for 1 min each, while BOLD signal changes were measured with 3T fMRI. The occurrence of positive and negative formal thought disorders was determined using standardized psychopathological assessments.

**Results:**

We found significant BOLD signal changes during spontaneous speech production in schizophrenia patients compared to healthy controls, particularly in the right hemispheric network. A *post-hoc* analysis showed that this right-hemispheric lateralization was mainly driven by activation during experimental rests. Furthermore, the TLI sum value in patients correlated negatively with BOLD signal changes in the right Rolandic operculum.

**Conclusions:**

Possible underlying factors for this inverse right-hemispheric lateralization of speech-associated areas are structural changes and transmitter system alterations, as well as a lack of neural downregulation in schizophrenia patients during rest periods due to dysfunctional executive functions. When examining spontaneous speech as the most natural form of language, other influencing factors, such as social cognition or emotional processing, should be considered. Our results indicate that future studies should consider group differences during rest, which might provide additional information typically covered in differential contrasts.

## 1 Introduction

Function-lesion observations in stroke and split-brain patients have revealed that some cognitive functions, such as speech processing, are lateralized into the left hemisphere, including the left superior temporal gyrus (STG) and inferior frontal gyrus (IFG) as speech-relevant areas (1, 2). In reviews and meta-analyses on structural MRI, a frequently replicated finding is the reduction of gray matter in the left STG in schizophrenia patients and their first-degree relatives, which suggests a genetic origin (3–6).

These volume reductions in the left STG correlate with formal thought disorder (FTD), a core symptom in patients with schizophrenia (3, 7, 8). FTD is an impairment in the structure of thoughts, speech, and communication and is commonly classified as positive or negative FTD. Positive FTD includes symptoms such as looseness of association, the use of neologisms, or pressured speech. Negative FTD includes symptoms like poverty of speech, mutism, or poverty of content (5, 9).

To date, neural processing of spontaneous speech production—as the most natural form of language—has only been examined in a few studies of patients with schizophrenia. Kircher and colleagues were the first to examine the neural processing of speech in six male native English-speaking right-handed patients with schizophrenia who had predominantly positive FTD (9). In their study, the patients and healthy control participants described Rorschach inkblots during scanning, and the number of spoken words was correlated with BOLD signal changes. In healthy control participants, the number of spoken words correlated positively with BOLD response in the left anterior STG, which was an expected and well-replicated finding; the left STG seems to be the physiological correlate of the main mental lexicon (9–11). The study’s main finding was that the number of words in patients with schizophrenia correlated positively with BOLD signal changes in the right STG. The authors interpreted this finding as an abnormal hemispheric lateralization during the production of spontaneous speech in patients with FTD—a conclusion that aligned with structural aberrations in the STG (9, 12, 13).

In contrast, Weiss and colleagues used a verbal fluency paradigm to examine seven German-speaking right-handed male patients with schizophrenia who exhibited acute psychotic symptoms using fMRI (14). Apart from benzodiazepines, which were not given on the day of the study, the patients received no other pharmacotherapy. On a behavioral level, the patients showed poorer performance in verbal fluency than the healthy control subjects. While the control group showed strong left hemispheric activation, the clinical group showed reduced language lateralization due to strong bilateral activation of Broca’s area. This reduced lateralization correlated negatively with the extent of hallucinations. The authors explained the reduced lateralization either by a lack of inhibition of the non-dominant language areas or by an increased bilateral cortical processing of language in patients with schizophrenia (14).

Based on the data from Kircher and colleagues, Matsumoto et al. used an event-related design to investigate the neuronal processing of pauses between sentences and filled pauses as markers of speech planning in patients with schizophrenia (15). Regarding filled pauses, they found activation of the left anterior STG, left insula, and right dorsolateral prefrontal cortex in the control group. The clinical group produced too few filled pauses, so no comparison with the control group was possible. However, during breaks between sentences, patients with FTD showed reduced activity in the left anterior temporal cortex and left insula compared to the controls. Since these areas are typically involved in speech planning and monitoring, the authors interpreted this finding as an impairment of discourse planning in patients with schizophrenia and FTD (15).

In previous studies that examined spontaneous speech processing in patients with schizophrenia, the patients were primarily in the acute phase of their illness. In the studies by Kircher et al. (9) and Matsumoto et al. (15), the male study population primarily exhibited FTD as a psychopathological symptom. The male study population examined by Weis and colleagues (14) was in an acute psychotic phase during the fMRI examination. Thus, the previous literature has failed to determine how language lateralization behaves in patients with schizophrenia in the chronic phase of their illness.

Since robust evidence for the correlation of right-hemispheric activation during spontaneous speech production in schizophrenia patients has yet to be provided, the aim of the present fMRI study is to examine BOLD signal changes during spontaneous speech in schizophrenia and schizophrenia spectrum disorder (SSD) patients compared to healthy control participants. To reflect a broader range of patients with schizophrenia, no special selection was made regarding sex or dominant psychopathological phenomena.

Referring to the studies by Kircher and Matsumoto, as well as previous evidence on structural changes in patients with schizophrenia, we hypothesized that the patients would activate a predominantly right-hemispheric speech network, including right-hemispheric lateralization of traditionally speech-relevant areas, such as the STG and IFG, due to structural and functional brain alterations. Second, we expected a positive correlation with the amount of positive FTD and BOLD signal changes in more right-hemispheric lateralized, traditionally speech-relevant areas.

## 2 Methods

### 2.1 Subjects and assessments

Fifteen patients with schizophrenia or SSD and 15 healthy controls were recruited via postings of in- and outpatients from the Department of Psychiatry and Psychotherapy at Philipps-University Marburg, Germany. DSM-IV criteria of the diagnosis were confirmed by a psychiatrist via a structural clinical interview. All patients had a diagnosis of schizophrenia or SSD (F20–F29) and received constant antipsychotic medication (for medication and diagnostic details, see **Supplementary Table A1** in the Supplementary Material). Healthy participants were free of psychiatric diseases in their medical history. The inclusion criteria were monolingual German as the mother language, right-handedness, and legal age. According to the declaration of Helsinki, all subjects gave written informed consent to participate in the study. The local ethics committee also approved the study (ethics proposal no. 37/10), and all participants were paid for their participation.

The schizophrenia group (SZ) consisted of 15 participants (13% women, *n* = 2) with a mean age of 38.8 years (SD = 14.33) and an average of 10.5 educational years (SD = 1.59). The healthy control group (HC) comprised 15 participants (20% women, *n* = 3) with a mean age of 38.8 years (SD = 10.57) and an average of 10.7 educational years (SD = 1.16). Participants in both groups were matched for age, sex, years of education, and level of education, with the latter dichotomized based on the presence of the university entrance qualification (for detailed information, see **Table 1**).

**Table 1:** Descriptive data for patients with schizophrenia (SZ) and healthy control participants (HC).

	SZ (*n* = 15)	HC (*n* = 15)	*p*-value
Age (years)	38.8 (SD: 14.33)	37.67 (SD: 10.57)	.991
Years of education	10.46 (SD: 1.59)	10.74 (SD: 1.16)	.512
Level of education	0.33 (SD: 0.48)	0.4 (SD: 0.5)	.576
Gender	13m; 2w	12m; 3w	.849
TLI pFTD sum	0.52 (SD: 0.57)	1.7 (SD: 3.49)	.965
TLI nFTD sum	2.45 (SD: 2.88)	1.97 (SD: 3.74)	.112
SAPS sum	11.2 (SD: 11.97)	–	–
Global pFTD sum	1.33 (SD: 1.25)	–	–
SANS sum	5.87 (SD: 6.13)	–	–
Global alogia	0.87 (SD: 0.88)	–	–

The functional imaging acquisition was performed by two experienced clinical psychologists. After scanning, speech was transcribed by a clinical linguist. An experienced psychiatrist, blind to the diagnosis, assessed and evaluated psychopathological phenomena for all participants using the Thought and Language Index (TLI) (16). In the clinical group, the sum value of the TLI was 0.52 (SD = 0.57) for positive FTD and 2.45 (SD = 2.88) for negative FTD. In the healthy control group, the sum value of the TLI was 1.7 (SD = 3.49) for positive FTD and 1.97 (SD = 3.74) for negative FTD. Within 48 h of scanning, psychopathological symptoms were also rated in patients by one of the clinical psychologists using the scales for the assessment of positive and negative symptoms (SAPS/SANS) (17, 18). We collected total values for SAPS (11.2, SD = 11.97), global positive FTD (1.33, SD = 1.25), SANS (5.87, SD = 6.13), and global alogia (0.87, SD = 0.88) for the clinical group (for detailed information regarding psychopathological symptom severity, see **Table 1** and **Supplementary Table A1** in the Supplementary Material).

### 2.2 Experimental material, task, and procedure

All participants were instructed to describe social situations in the MRI scanner, where at least two persons were pictured using eight pictures from the Thematic Apperception Test (TAT) (19). The exact instruction for the participants was as follows: “Please describe in as much detail as possible what you see in the picture and what could be going on there.” The pictures were presented on a monitor (aspect ratio: 16:9) behind the scanner and mirrored via a display approximately 15 cm from the participants as they lay in the MRI scanner. The sequence of the pictures was randomized to avoid sequence effects.

At the beginning of each trial, a fixation cross was presented for 5 ms. After every picture depiction, a rhombus was presented on the screen for a duration of exactly 15 s. This duration of 15 s, which was equal for all participants and was defined as *experimental rest*, was the contrast of interest. The overall duration of the spontaneous speech paradigm was 10 min (eight pictures of 60 s + eight experimental rest periods of 15 s). Furthermore, every participant displayed an individual behavior regarding rests during speech blocks, defined as *self-chosen rests*, which were considered a contrast of no interest. Regarding previous evidence for the German language, we considered self-chosen rests during speech blocks of at least 100 ms (20).

### 2.3 Behavioral data acquisition and analysis

Participants’ speech was recorded during the entire scanning session using a noise-reducing microphone system (FOMRI-II, Optoacoustics Ltd., Or-Yehuda, Israel) including a dual adaptive filter system (21) that subtracted the reference input (background MRI noise) from the source input (speech signal). The microphone was fixed at the head coil and wired to the sound filter box. The output port was directly wired to the audio in-line plug of the notebook sound card.

The digitally recorded audio files (Audacity, version 1.2.6, Softonic International S.L.) were saved in 16-bit WAV format and transcribed into text files using the F4 plus transcription software (version 5.10.x, Dr. Dresing & Pehl GmbH, Marburg, Germany). Contrary to the F4 transcription guidelines, hesitations (like “ehm,” “uh”), breakup of words, and self-chosen rests were transcribed for the correct presentation of FTD. Regarding previous evidence for the German language, we considered self-chosen rests of at least 100 ms (20). We considered every realized word and did not distinguish between word types and tokens (22).

### 2.4 FMRI data acquisition and analysis

#### 2.4.1 fMRI data acquisition

Imaging was performed on a 3T-MRI Magnetom Trio Trim scanner (Siemens, Erlangen, Germany). Since spontaneous speech production during data collection in the MRI can lead to articulation-related head movements, thereby causing artifacts, the participants’ heads were fixated using standardized foam pads by an experienced medical–technical radiology assistant from the brain imaging working group to minimize speech-related head motion. T1-weighted, high-resolution anatomical images were acquired for each participant. Functional data were acquired using a T2-weighted echo planar image (EPI) [repetition time (TR) = 2,000 ms; echo time (TE) = 30 ms; flip angle = 90°]. The volume included 33 transversal slices [slice thickness = 3.6 mm; interslice gap = 0.36 mm; field of view (FoV) = 230 mm; voxel resolution = 3.6 mm²]. During the functional run, 300 volumes were acquired.

#### 2.4.2 fMRI data analysis

##### 2.4.2.1 Preprocessing

The data were analyzed using the standard routines of the SPM8 software. Speech transcripts during scanning were divided into sections of 20 s. Then, the number of every realized word per 20-s block was counted. The experiment began with the presentation of the first fixation cross to guide attention. Subsequently, the first image of the TAT was shown. Images were realigned to the first image, as uncorrected subject motion can produce type I or type II errors (23, 24). The data analysis and correction procedure were conducted analogously to previous studies on spontaneous speech acquisition in fMRI by Kircher (9) and the brain-imaging working group (22, 25).

Before beginning the first-level analysis, a visual inspection of the movement parameters (*x*, *y*, *z*, roll, pitch, jaw) was performed to ensure movement did not exceed 6 mm. Moreover, no “spiking” phenomena were observed. In a further step, movement parameters (realignment data) were individually partialized out in the SPM first-level analysis, as these were included as multiple regressors in the design matrix. Therefore, the effect of speech production on movement was considered in the analyses.

Afterward, realigned images were normalized into standard stereotaxic anatomical space using the transformation matrix (mean image) calculated from the first EPI scan for each subject and the EPI template created by the Montreal Neurological Institute (MNI). The normalized data (resliced voxel size: 2 mm³) were then smoothed with an 8-mm Gaussian kernel to increase the signal-to-noise ratio and compensate for intersubject variance in brain anatomy.

##### 2.4.2.2 First-level analysis

Using preprocessed data, the BOLD response was analyzed for every participant. Considering the respective group affiliation (SZ for the patient group and HC for healthy control subjects) and onset times of realized words and pauses (i.e., rests) during fluent speech, eight regressors were defined to perform directed T-statistics. Speech production and rest periods were defined for each participant, as were self-chosen rest periods. To consider the number of spoken words, a parametric regressor was defined for the speech production condition (in total, three conditions and one parametric regressor per subject). Thus, we defined the following:

- *SZ block* and *HC block*: 20-s speech production block for patients and healthy control participants.
- *SZ* and *HC block words*: the number of words produced per 20-s block for each group as a parametrical regressor.
- *SZ* and *HC experimental rest*: experimental rests given by the study design.
- *SZ* and *HC rest self:* self-chosen rests during fluent speech with a duration of at least 100 ms.

The duration of the experimental (*SZ* and *HC duration rest exp*) and self-chosen rests (*SZ* and *HC duration rest self*) was also evaluated for the correct representation of the baseline speech but was defined as contrasts of no interest.

##### 2.4.2.3 Second-level analysis

Next, using the results of the first statistical analysis, analyses of variance were conducted to detect significant BOLD signal changes within and between the two groups. To elucidate general activity differences between the groups, a whole-brain analysis was conducted. Using the coordinates for STG and IFG of this analysis, eigenvariates from peak voxels (4-mm sphere) of the STG and IFG (Rolandic operculum) were extracted using the VOI function of SPM. Our major contrast of interest—defined in a full factorial design—was the interaction of the speech production versus the experimental rest of the clinical group in comparison to healthy control participants. To enable comparison with the data of Kircher and colleagues, we chose a previously defined statistical threshold for the fMRI analysis of *p* <.001. Only results containing at least 20 coherent voxels were considered (for details, see **Supplementary Table A2**; for FWE correction, see **Supplementary Figure A1**).

In our correlation analysis, we extracted contrast estimates from speech-relevant areas for the clinical group, particularly peak voxels from the right STG and IFG (right Rolandic operculum), with a sphere radius of 4 mm. The neural activity during speech production was identified in a parametric analysis considering the amount of speech, operationalized as the number of words per 20-s block. The activation explained by the number of words was then correlated with psychopathologic assessments. To monitor the severity of positive and negative FTD, we used the TLI’s sum score for positive and negative FTD (“sum of pPFTD” and “sum of nFTD”) (16) as well as the sum and subscales of SAPS (“sum pFTD,” “global pFTD”) and SANS (“sum of nFTD/alogia,” “global alogia”) (17, 18). The analysis of correlation was conducted using Spearman’s rank correlation (SPSS Software; IBM-SPSS-Software, 2020) and corrected for multiple-comparison testing using Bonferroni correction.

## 3 Results

### 3.1 Whole-brain analysis

Initially, independent of the activation direction, significant neural activation differences were found during spontaneous speech production of healthy control participants (HC) compared to that of patients with schizophrenia (SZ) relative to experimental rests (*[HC speech > HC exp. rest] > [SZ speech > SZ exp. rest]*). In the clinical group, right-hemispheric BOLD signal changes were found in the superior frontal gyrus, medial frontal gyrus, and IFG (particularly the Rolandic operculum and orbital part); medial temporal pole; STG; hippocampus; and cerebellum. Furthermore, relative to the patients, the healthy control participants showed left-hemispheric BOLD signal changes in the postcentral gyrus and left hippocampus (see **Table 2**; for details, see **Supplementary Table A2**). These differences in activity were not observed in the opposite contrast *(*[SZ speech > SZ exp. rest] > [HC speech > HC exp. rest]*)*.

**Table 2:** Significant activations in the whole-brain analysis for the contrast [(HC speech > HC exp. rest) > (SZ speech > SZ exp. rest)].

Anatomical region		Coordinates				
	Hemisphere	*x*	*y*	*z*	*F*	No. of voxels
**Postcentral gyrus**	L	−64	−2	20	21.94	99
**Inferior frontal gyrus (Rolandic operculum)**	R	60	4	16	20.28	203
**Medial temporal pole**	R	40	12	−42	17.11	30
**Hippocampus**	L	−32	−32	−8	16.92	26
**Superior temporal gyrus**	R	58	4	−14	16.43	24
**Middle frontal gyrus**	R	50	30	32	16.31	37
**Hippocampus**	R	40	−34	−2	15.61	41
**Inferior frontal gyrus (orbital part)**	R	30	36	−10	15.13	29
**Cerebellum**	R	24	−62	−24	14.93	93
**Superior temporal gyrus**	R	52	−24	0	13.19	23

**Figure 1:**
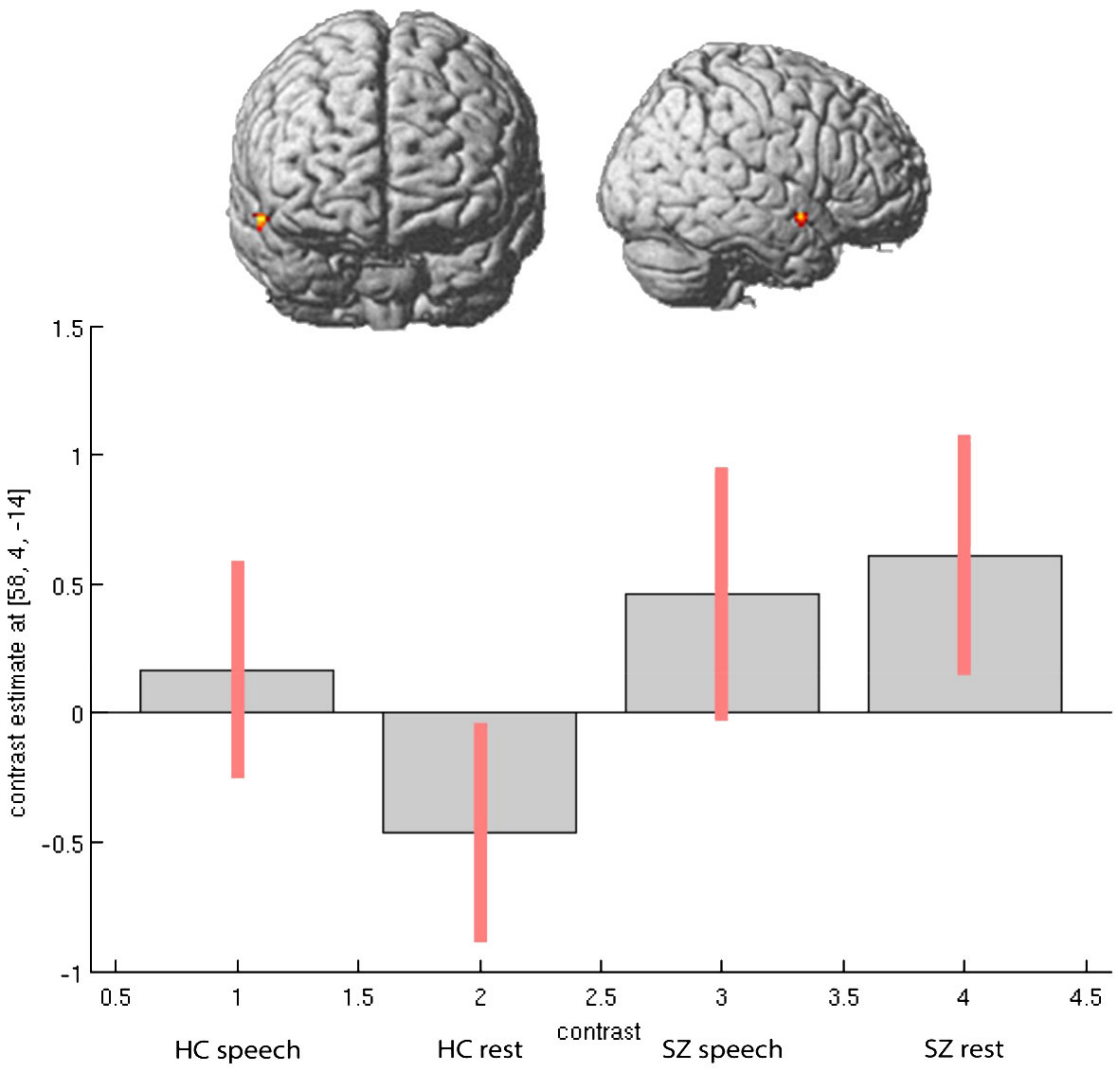
Activation differences in right STG (coordinates- 58, 4, -14) within groups and between groups during spontaneous speech production and experimental rests.

**Figure 2:**
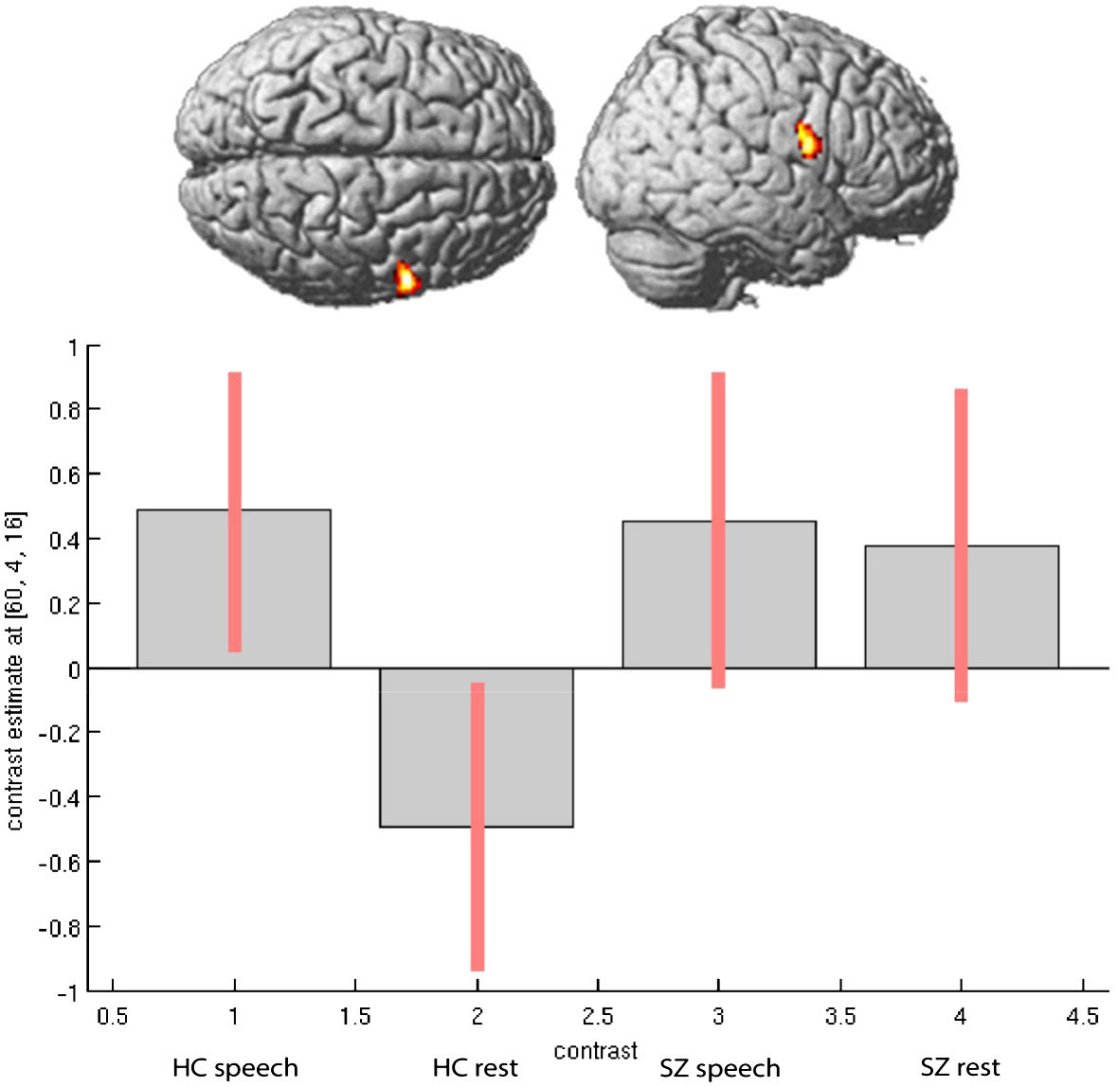
Activation differences in right Rolandic Operculum (coordinates- 60, 4, 16) within groups and between groups during spontaneous speech production and experimental rests.

In the second step, we considered the direction of activation differences between the groups in speech-relevant areas, including the right STG and right IFG (Rolandic operculum). Particularly in the region of the right STG, patients showed increased neural activation during experimental rests relative to the healthy control participants (see **Figure 1**). In the right IFG, particularly in the Rolandic operculum, patients showed opposite neural activation patterns to the healthy control participants: During experimental rests, the control group showed reduced neural activity, whereas the clinical group showed increased neural activity (see **Figure 2**). For both areas, no significant effect with antipsychotic medication, operationalized as chlorpromazine equivalent, was observed (for detailed information, see **Supplementary Table A3**).

**Figure 3:**
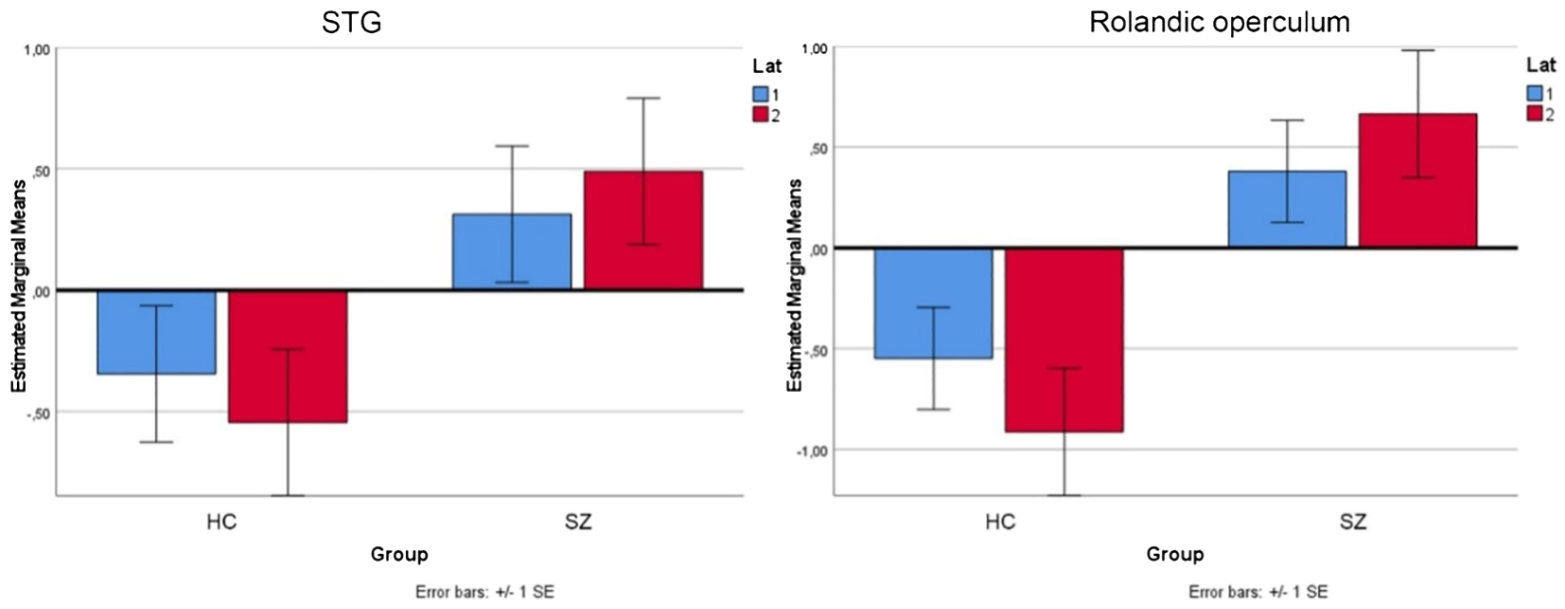
Repeated measure ANOVA for the area of STG and Rolandic operculum for both groups (HC, SZ) and experimental rests in the left (lat 1=blue) and right (lat 2=red) hemisphere.

As mentioned above, we observed increased activation in the right STG and right Rolandic operculum in the patients during experimental rests. To show that the experimental activation was actually stronger in the right-hemispheric network than in the left one, we performed a repeated measure ANOVA as a *post-hoc* analysis with the extracted contrast-estimates (eigenvariates) of homolog regions from the left and right hemispheres in the area of the Rolandic operculum and STG for both groups. We found significant group, task, and laterality interactions. As shown in **Figure 3**, the effect of lateralization was mainly driven by activation during experimental rests in the areas of interest, with significant differences between the clinical groups (STG: *F* = 4.56, *p* <.042; Rol. operc.: *F* = 10.373, *p* <.003).

### 3.2 Peak voxel and correlation analysis

In our correlation analysis, we extracted contrast estimates from speech-relevant areas for the patients, particularly peak voxels from the right STG and IFG (area of the right Rolandic operculum) with a sphere radius of 4 mm. The neural activity during speech production, which was correlated with the number of words per block (included as a parametrical regressor in the model), was then correlated with psychopathological assessments. The amount of activity in the right STG (4-mm sphere), explained by the number of spoken words, correlated negatively with the SAPS sum scale “global PFTD” (rho: −0.521, *p* = .046). However, this effect was not robust to multiple testing. No correlation was found between the amount of activity in the right STG (4-mm sphere) and antipsychotic medication (rho: −0.317, *p* = .25; for detailed information, see **Supplementary Table A3**).

The amount of activity in the right Rolandic operculum (4-mm sphere), explained by the number of spoken words, correlated negatively with the TLI subscale “PFTD sum” (rho: −0.604, *p* = .017; see **Table 3**). This effect was stable after multiple testing. No significant correlation was found between the amount of activity in the right Rolandic operculum (4-mm sphere) and antipsychotic medication (rho: −0.97, *p* = .731; see **Supplementary Table A3**).

**Table 3:** Correlation between measures of psychopathology (TLI, SAPS/SANS) and the variance in BOLD signal accounted for by the number of words per 20-s speech block in the right Rolandic operculum in SZ.

SZ block words	rho	*p*-value
TLI PFTD sum	−0.604	.017*
TLI NFTD sum	−0.092	.745
SAPS sum	−0.133	.636
Global PFTD	−0.229	.411
SANS sum	0.312	.258
Global alogia	−0.067	.812

*Indicates significance, corrected for multiple testing.

## 4 Discussion

In our study, patients with schizophrenia and healthy participants described pictures from the TAT, while BOLD signal changes were measured using 3T fMRI. We found significant BOLD signal changes during spontaneous speech production compared to experimental rests in patients with schizophrenia compared to healthy participants, particularly in the right-hemispheric network. A *post-hoc* analysis showed that this effect of right-hemispheric lateralization was mainly driven by activation during experimental rests in patients. Furthermore, we examined the neural correlates of the mental lexicon, operationalized as the number of words spoken per 20-s epoch, during spontaneous, natural speech in schizophrenia patients and correlated this with the severity of FTD. The amount of activity in the right IFG, explained by the number of spoken words, correlated negatively with positive FTD.

### 4.1 Right-hemispheric neuronal network in schizophrenia

The whole-brain analysis revealed significant neural activation differences between the groups. During spontaneous speech production, patients showed BOLD signal changes in a right-hemispheric network in comparison to healthy control participants, particularly in the superior frontal gyrus, medial frontal gyrus, and IFG (especially the orbital part and Rolandic operculum); STG; medial temporal pole; hippocampus; and cerebellum. Furthermore, in contrast to the control participants, the patients showed left-hemispheric BOLD signal changes in the postcentral gyrus and hippocampus. Therefore, the clinical group showed a reversed lateralization of areas *within* and *outside* of the traditional language-related network. These findings aligned with previous fMRI research in patients with schizophrenia (3, 5, 7, 9, 26).

One reason for the predominantly right-hemispheric lateralization in the IFG and STG could be structural volume reductions in the homologous left hemisphere areas (3–6, 8, 27, 28). Another reason could be the task of spontaneous speech elicitation on which this work is based: The TAT (19) partially depicts ambiguous everyday situations. In other words, the neuronal activation during the elicitation of spontaneous speech by the TAT can also be influenced by the effects of social cognition (29).

Moreover, patients also presented activation differences in areas *outside* the classical language-related network, such as the right cerebellum, which plays an important role in auditory self-monitoring as well as articulatory speech planning (3). These functions are often impaired in schizophrenia patients (30–32).

### 4.2 Lateralization of IFG and STG

Patients showed an increase in neural activity during rest conditions relative to speech in the STG and IFG lateralized to the right hemisphere, whereas the healthy participants showed clear activation differences between rest and speech. A *post-hoc* analysis of this unexpected finding showed that the effect of right-hemispheric lateralization was mainly driven by activation during experimental rests in schizophrenia patients. This finding suggested a general overactivation of the right-hemispheric language areas in schizophrenia patients.

Models of semantic priming or mental lexicon (10, 33, 34) assume a main lexicon in the left STG with rapid, targeted activation of semantically closely related terms, as well as an additional lexicon in the right STG, which ensures slower and more widespread activation of semantically more distant terms. The latter is necessary for the reception of sentences and decoding of ambiguity (35). In the context of schizophrenia, ambiguity means that patients with FTD experience challenges with the pragmatic level of speech. These problems may manifest in interpreting daily situations and their social implications incorrectly due to a lack of transferring the intended meaning or abstract semantic content of metaphoric expression (36). In an event-related fMRI study, Rapp and colleagues examined 15 female patients with schizophrenia in comparison to healthy control participants. The participants had to identify whether target sentences had ironic, literal, or meaningless content. BOLD signal change was decreased in patients during irony comprehension, particularly in the right temporal and posterior medial prefrontal regions, as well as the left insular regions, whereas the left parahippocampal gyrus showed an increased activation relative to healthy participants (36). In the TAT used for spontaneous speech elicitation in the current study, some pictures depicted ambiguous social situations. For this reason, the pragmatic level, which is often disturbed in patients with schizophrenia, may have influenced lateralization.

The lack of downregulation in the right STG during experimental rests could be explained by word retrieval, which is more unprecise in schizophrenia patients due to extended, increased activation of the additional lexicon (9). Regarding the right IFG, the Rolandic operculum is both an element of the opercular part of Broca’s area and a component of the precentral gyrus (37). Therefore, other functions in this area may influence experimental rests, such as processing emotions or sensory information (38, 39), which only had been relevant for the clinical group but were not depicted by the present paradigm.

Various studies have shown that schizophrenia patients with persisting symptoms have heightened emotional recognition deficits (40, 41), particularly in recognizing facial emotions correlating with negative symptoms (42). As a component of the precentral gyrus, the Rolandic operculum plays an important role in emotional processing through the fronto-limbic structures (38). Potvin and colleagues investigated 39 patients with schizophrenia as well as healthy control participants who rated emotionally positive, negative, and neutral images during fMRI scanning (40). They found that patients with schizophrenia gave emotionally neutral situations more emotional meaning than controls. Both groups showed significant activations in the dorso-medial prefrontal cortex (dmPFC) and the bilateral amygdala. However, in contrast to the healthy control participants, the Granger connectivity from the right amygdala to the dmPFC was reduced in patients during the neutral and negative conditions. Furthermore, the Granger connectivity from the left amygdala to the dmPFC was reduced in patients during the positive condition. This finding of reduced connectivity between the left and right hemisphere amygdala to the dorso-medial prefrontal gyrus may explain why patients with schizophrenia attach emotional meaning to emotionally irrelevant (i.e., neutral) stimuli (40). Since all images in the TAT used in the present study depicted people with different facial expressions, the influence of misinterpretation of emotional stimuli by the patient group must also be considered.

For right STG and right IFG, the observed lack of downregulation during experimental rests in patients may also have been due to dysfunctional executive functions. Selecting convenient thoughts in a special context and suppressing non-convenient thoughts are fundamental for coherent speech. However, this ability is often reduced in schizophrenia patients and may lead to an ongoing neural activation during experimental rests (11). Therefore, this should be considered in future studies on spontaneous speech in patients with schizophrenia; test procedures to assess executive functions, such as the Trail Making Test (TMT) (43), should be used.

Regarding the lateralization of language, the gender distribution of the patient population should also be considered. Our clinical group consisted of 13 male and two female patients. Gender differences are a frequently replicated finding in schizophrenia research (44). While women present the first symptoms of the disease an average of 5 five years later than men (45), the severity of these symptoms and the response to pharmacotherapy also differ (44). For example, women often show positive symptoms, while men tend to show negative symptoms (46).

In an fMRI study, Sommer and colleagues examined the language lateralization of female patients with schizophrenia compared to healthy subjects (47). In the clinical group, language lateralization in left hemisphere areas was lower compared to the control group. However, activation of the right hemisphere language areas was increased. The group of female schizophrenia patients was then compared with male schizophrenia patients (48). The male group also showed less left-sided language lateralization compared to healthy individuals, as well as increased activation on the right side. Therefore, no significant group difference in language lateralization was found between the clinical groups (47). Despite known gender-specific peculiarities in patients with schizophrenia, we do not assume any influences of gender-specific differences in language lateralization, based upon these findings from Sommer.

### 4.3 Lateralization and psychopathology

To achieve the second aim of this fMRI study, the variance in BOLD signal accounted for by the number of words per 20-s speech block was correlated with measures of psychopathology (TLI, SAPS/SANS). The amount of activity in the right Rolandic operculum, explained by the number of spoken words, correlated negatively with the severity of positive FTD measured with the TLI subscale “PFTD sum”.

Although the patients did not differ from the healthy control subjects in terms of the severity of FTD at the group level, there was a significant group difference at the neuronal level. At this point, the existing pharmacotherapy of the patients must be considered: Atypical antipsychotics, such as clozapine or quetiapine, bind to and block the dopamine receptor (D2-R) with a lower affinity than typical antipsychotics, such as haloperidol, which cause a strong D2-R blockade. In a behavioral design, deBoer and colleagues compared the spontaneous speech production of patients receiving antipsychotics with high and low affinity for the D2-R (49). They found that patients who used antipsychotics with high D2-R occupancy showed more pronounced negative speech disorders, such as increased pauses and shortened utterance length, compared to patients who used antipsychotics with low D2-R occupancy (49).

Regarding the influence of antipsychotic medication on the lateralization of language, Hwang and colleagues investigated 21 therapy-naive patients with psychotic symptoms before and after 6 weeks of treatment with aripiprazole (50). During fMRI scanning, patients and healthy control participants completed a semantic decision paradigm. This task was repeated after 6 weeks of treatment with the atypical antipsychotic. After treatment, the patients showed an increased left-hemispheric lateralization index, particularly in the IFG, which was due to decreased activity in the right-hemispheric IFG. This improved lateralization was positively correlated with verbal fluency. The authors explained the improved semantic performance by the increased left-hemisphere lateralization caused by aripiprazole (50). Regarding the stability of functional language lateralization over time in schizophrenia patients, Razafimandimby and colleagues investigated 10 patients with schizophrenia in comparison to healthy control participants in two fMRI sessions at an interval of 21 months (51). All patients were in a stable chronic phase of their illness with no psychotic exacerbation during the study period and were regularly treated with either typical or atypical antipsychotics. The authors found significantly reduced left hemisphere lateralization in the clinical group compared to the control group, especially in the so-called semantic areas of interest. Since this effect was not influenced by the study period, the specific task, or the presence of psychotic symptoms, the authors advocated a special organization of language in patients with schizophrenia (51).

In the present study, all patients were also in the chronic phase of their disease and received different antipsychotic medications, as well as co-medication, comparable to the abovementioned study. Only three of the patients received aripiprazole, and only one received it in monotherapy. Nevertheless, an effect of antipsychotic medication on the lateralization of language seems possible and should be re-examined in future studies on spontaneous speech in patients with schizophrenia using a homogeneous antipsychotic medication. However, regarding chlorpromazine equivalent doses (see **Supplementary Table A3** in the Supplementary Material), medication did not influence the within-patient correlational analyses. Comparable to the study by Razafimandimby and colleagues, these results suggest an abnormal neural organization of language in patients with schizophrenia that appears to be independent of the duration of the illness (51).

Because of its topography in the human brain, the right Rolandic operculum is not only relevant in speech processing but also in processing emotion (39) and sensory information (38, 52). Due to this role, this area might influence symptoms like delusions (47, 48), depression, or anxiety (39). Future studies should consider the influence of these additional psychopathological symptoms. Our aim was to reflect a broader range of schizophrenia patients, so we avoided highly selected participants regarding the single phenomenon of FTD.

## 5 Limitations

This study had some limitations. First, despite the small number of patients, previous studies have suggested a large effect size for group differences in verbal production (9, 11, 15) (*d* = 1.453). Therefore, the current sample size should be sufficient to obtain valid results. Nevertheless, future studies should select a larger sample of only people suffering from schizophrenia. Second, all patients received antipsychotic medication with either atypical or typical antipsychotics, as well as co-medication. The utilization of antipsychotic medication and co-medication might have influenced the transmitter systems relevant to lateralization. Third, the task of this study should be considered: Since spontaneous speech was examined as the most natural form of language in a picture description paradigm, the influence of additional factors, such as emotional processing or social cognition, cannot be ruled out. Finally, speech production might be related to head movement, which could affect data quality, although our data were collected in rest periods, and movement was not present.

## 6 Conclusions

Using a case–control design, this pilot study examined spontaneous speech in a German-speaking group of patients with schizophrenia using fMRI. Traditional speech-relevant areas were lateralized inversely in the right hemisphere in the clinical group. Furthermore, the amount of activity in the right Rolandic operculum, as explained by the number of spoken words, correlated negatively with the severity of positive FTD. These results aligned with those of previous studies that examined spontaneous speech in patients with schizophrenia as the most natural form of language (9, 11, 15). Additionally, the current study showed that disruptions at certain linguistic levels, such as semantics or pragmatics as well as altered interpretation of emotional stimuli due to the used material, can influence the neural processing of spontaneous speech in patients with schizophrenia.

A *post-hoc* analysis showed that the effect of right-hemispheric lateralization was mainly driven by activation during experimental rests, possibly due to dysfunctional executive functions. Further studies on spontaneous speech processing in schizophrenia patients should include larger samples and focus on pause behavior as a component of the speaking–thinking process. This can contribute to an improved understanding of FTD in patients with schizophrenia.

## Data availability statement

The raw data supporting the conclusions of this article will be made available by the authors, without undue reservation.

## Ethics statement

The studies involving humans were approved by the local ethics committee of Phillips-University Marburg. The studies were conducted in accordance with the local legislation and institutional requirements. The participants provided their written informed consent to participate in this study. Written informed consent was obtained from the individual(s) for the publication of any potentially identifiable images or data included in this article.

## Author contributions

WH: Conceptualization, Writing – original draft, Data curation. P-ET: Writing – review & editing. AN: Conceptualization, Writing – review & editing, Funding acquisition. BS: Conceptualization, Writing – original draft, Writing – review & editing, Methodology, Supervision.

## In memoriam

This paper is dedicated to the memory of Arne Nagels, who passed away on April 9th, 2024.
