# Supplementary material for: Neural activation during natural speech and rests in patients with schizophrenia and schizophrenia spectrum disorders—an fMRI pilot trial
Source: Front Psychiatry. 2024 Jun 13;15:1402818. doi: 10.3389/fpsyt.2024.1402818 (PMC11210388; doi:10.3389/fpsyt.2024.1402818)

Table A 1: Overview of the clinical group (SZ) regarding diagnosis (ICD-10), antipsychotic medication, chlorpromazine (CPZ) equivalent and further psychotropic medication in mg per day (mg/d) as well as psychopathological assessments

| **SZ participant** | **Diagnosis** | **Antipsychotic Medication (mg/d)** | **CPZ^T^ equivalent (mg/d)** | **Additive psychotropic drugs (mg/d)** | **TLI PFTD sum** | **TLI NFTD sum** | **SAPS Sum** | **Global PFTD** | **SANS Sum** | **Global alogia** |
| --- | --- | --- | --- | --- | --- | --- | --- | --- | --- | --- |
| 1 | F21.0 | 4 mg risperidone^A^ | 240 mg | none | 0.25 | 0.75 | 36 | 4 | 6 | 1 |
| 2 | F20.0 | yes (but unknown) | - | unknown | 0 | 1 | 8 | 2 | 10 | 1 |
| 3 | F25.2 | 5 mg risperidone^A^ | 300 mg | 900 mg lithium,  60 mg citalopram | 0 | 0 | 0 | 0 | 6 | 1 |
| 4 | F20.0 | 300 mg clozapine^A^ | 300 mg | 25 mg fluvoxamine | 0 | 0 | 20 | 2 | 0 | 0 |
| 5 | F20.0 | 25 mg olanzapine^A^,  4 mg risperidone^A^ | 750 mg  240 mg | none | 0.5 | 0.25 | 28 | 2 | 12 | 2 |
| 6 | F20.0 | 650 mg clozapine^A^ | 650 mg | 50 mg fluvoxamine | 0.25 | 0.25 | 10 | 1 | 0 | 0 |
| 7 | F20.0 | 250 mg clozapine^A^  800 mg quetiapine^A^  10 mg aripiprazole^A^ | 250 mg  600 mg  200 mg | 25 mg fluvoxamine | 1.75 | 5.5 | 12 | 2 | 6 | 1 |
| 8 | F20.1 | 800 mg quetiapine^A^ retard,  4 mg risperidone^A^ | 600 mg  240 mg | 50 mg citalopram | 0.75 | 1.75 | 24 | 3 | 4 | 1 |
| 9 | F20.0 | 800 mg quetiapine retard^A^,  olanzapine^A^, (dose unknown) | 600 mg | none | 0.75 | 0 | 0 | 0 | 6 | 1 |
| 10 | F20.0 | risperidone (dose unknown), quetiapine^A^ (dose unknown) |  | none | 1 | 1.75 | 4 | 0 | 0 | 0 |
| 11 | F20.0 | 15 mg aripiprazole^A^ | 300 mg | none | 0.5 | 3 | 0 | 0 | 0 | 0 |
| 12 | F20.0 | 150 mg clozapine^A^,  15 mg aripiprazole^A^ | 150 mg  300 mg | none | 0 | 3 | 0 | 0 | 0 | 0 |
| 13 | F20.0 | clozapine^A^ (dose unknown),  benperidol^T^ (dose unknown) |  |  | 0.25 | 5.25 | 14 | 2 | 24 | 3 |
| 14 | F20.0 | 210 mg/month olanzapine pamoate^A^ | 63 mg (depot) | none | 0.5 | 3.25 | 0 | 0 | 6 | 0 |
| 15 | F20.0 | 10 mg olanzapine^A^ (10 mg),  100 mg flupentixol depot^T^ | 300 mg  no equivalent | none | 1.75 | 11 | 12 | 2 | 8 | 2 |

^T^= Typical antipsychotics. Substance group within the antipsychotics that have an antipsychotic effect primarily through D₂ antagonism.

^A^= Atypical antipsychotics. Substance group within the antipsychotics that, in addition to the D_2-_receptor, also have an antipsychotic effect via other receptors (D_1_, D^3^, 5-HT and others). Due to the lower D_2_ antagonism, there are significantly fewer or no extrapyramidal motor side effects.

Table A 2: Whole-brain analysis for the condition SZ vs. HC speech vs. experimental rests (Height threshold F=11.421, p<0.001 (uncorr.), extent threshold k=20 voxels)

| *Set-level* | |  | *cluster-level* | | | |  | *peak-level* | | | | |  | mm | mm | mm |
| --- | --- | --- | --- | --- | --- | --- | --- | --- | --- | --- | --- | --- | --- | --- | --- | --- |
| *p* | *c* |  | *p*_FWE-corr_ | *q*_FDR-corr_ | *k*_E_ | *p*_uncorr_ |  | *p*_FWE-corr_ | *q*_FDR-corr_ | *F* | *(Z_≡_)* | *p*_uncorr_ |  |  |  |  |
| **< 0.001** | **14** |  |  |  | **1687** |  |  | **0.003** | **0.038** | **31.61** | **5.14** | **0.000** |  | **20** | **30** | **20** |
|  |  |  |  |  |  |  |  | 0.126 | 0.274 | 20.81 | 4.20 | 0.000 |  | 30 | 44 | 14 |
|  |  |  |  |  |  |  |  | 0.150 | 0.274 | 20.31 | 4.15 | 0.000 |  | 18 | 88 | 32 |
|  |  |  |  |  | **99** |  |  | **0.085** | **0.274** | **21.94** | **4.32** | **0.000** |  | **-64** | **-2** | **20** |
|  |  |  |  |  | **990** |  |  | **0.090** | **0.274** | **21.77** | **4.30** | **0.000** |  | **-22** | **28** | **22** |
|  |  |  |  |  |  |  |  | 0.149 | 0.274 | 20.34 | 4.16 | 0.000 |  | -30 | 22 | 22 |
|  |  |  |  |  |  |  |  | 0.240 | 0.239 | 18.91 | 4.01 | 0.000 |  | -18 | 8 | 20 |
|  |  |  |  |  | **203** |  |  | **0.152** | **0.274** | **20.28** | **4.15** | **0.000** |  | **60** | **4** | **16** |
|  |  |  |  |  | **30** |  |  | **0.416** | **0.336** | **17.11** | **3.81** | **0.000** |  | **40** | **12** | **-42** |
|  |  |  |  |  | **26** |  |  | **0.439** | **0.336** | **16.92** | **3.79** | **0.000** |  | **-32** | **-32** | **-8** |
|  |  |  |  |  | **185** |  |  | **0.440** | **0.336** | **16.91** | **3.79** | **0.000** |  | **-42** | **-12** | **26** |
|  |  |  |  |  |  |  |  | 0.684 | 0.478 | 15.08 | 3.58 | 0.000 |  | -38 | -6 | 18 |
|  |  |  |  |  |  |  |  | 0.957 | 0.802 | 12.38 | 3.32 | 0.000 |  | -46 | -20 | 42 |
|  |  |  |  |  | **24** |  |  | **0.501** | **0.373** | **16.43** | **3.74** | **0.000** |  | **58** | **4** | **-14** |
|  |  |  |  |  | **37** |  |  | **0.517** | **0.377** | **16.31** | **3.72** | **0.000** |  | **50** | **30** | **32** |
|  |  |  |  |  | **41** |  |  | **0.611** | **0.428** | **15.61** | **3.64** | **0.000** |  | **34** | **-34** | **2** |
|  |  |  |  |  | **29** |  |  | **0.676** | **0.478** | **15.13** | **3.58** | **0.000** |  | **30** | **36** | **-10** |
|  |  |  |  |  | **93** |  |  | **0.704** | **0.481** | **14.93** | **3.56** | **0.000** |  | **24** | **-62** | **-24** |
|  |  |  |  |  |  |  |  | 0.921 | 0.694 | 12.96 | 3.30 | 0.000 |  | 14 | -68 | -16 |
|  |  |  |  |  | **27** |  |  | **0.891** | **0.677** | **13.32** | **3.35** | **0.000** |  | **24** | **-22** | **24** |
|  |  |  |  |  | **23** |  |  | **0.903** | **0.688** | **13.19** | **3.34** | **0.000** |  | **52** | **-24** | **0** |

Table A 3: Chlorpromazine equivalent dose in SZ and correlation to imaging results. Data is corrected for multiple testing.

| **CPZ equivalent dose (mg per day)** | |  | |  | |
| --- | --- | --- | --- | --- | --- |
|  | **rho** | | **p** | |  |
| Whole-brain analysis |  | |  | |  |
| R STG SZ block | 0.345 | | .207 | |  |
| R Rolandic operculum SZ block | 0.478 | | .710 | |  |
| R STG exp pause | 0.610 | | .829 | |  |
| R Rolandic operculum exp pause | 0.900 | | .750 | |  |
| Peak voxel analysis |  | |  | |  |
| R STG (4-mm sph) SZ block words | -0.317 | | .250 | |  |
| R Rolandic operculum (4-mm sph) SZ block words | -0.970 | | .731 | |  |

Figure A 1: Illustration of the results corrected for multiple comparisons at the FWE cluster level for our main contrast of interest: group (SZ vs. HC) by task (speech vs. experimental rest) interaction. At the cluster level of 552 voxels, two clusters survived the cluster correction


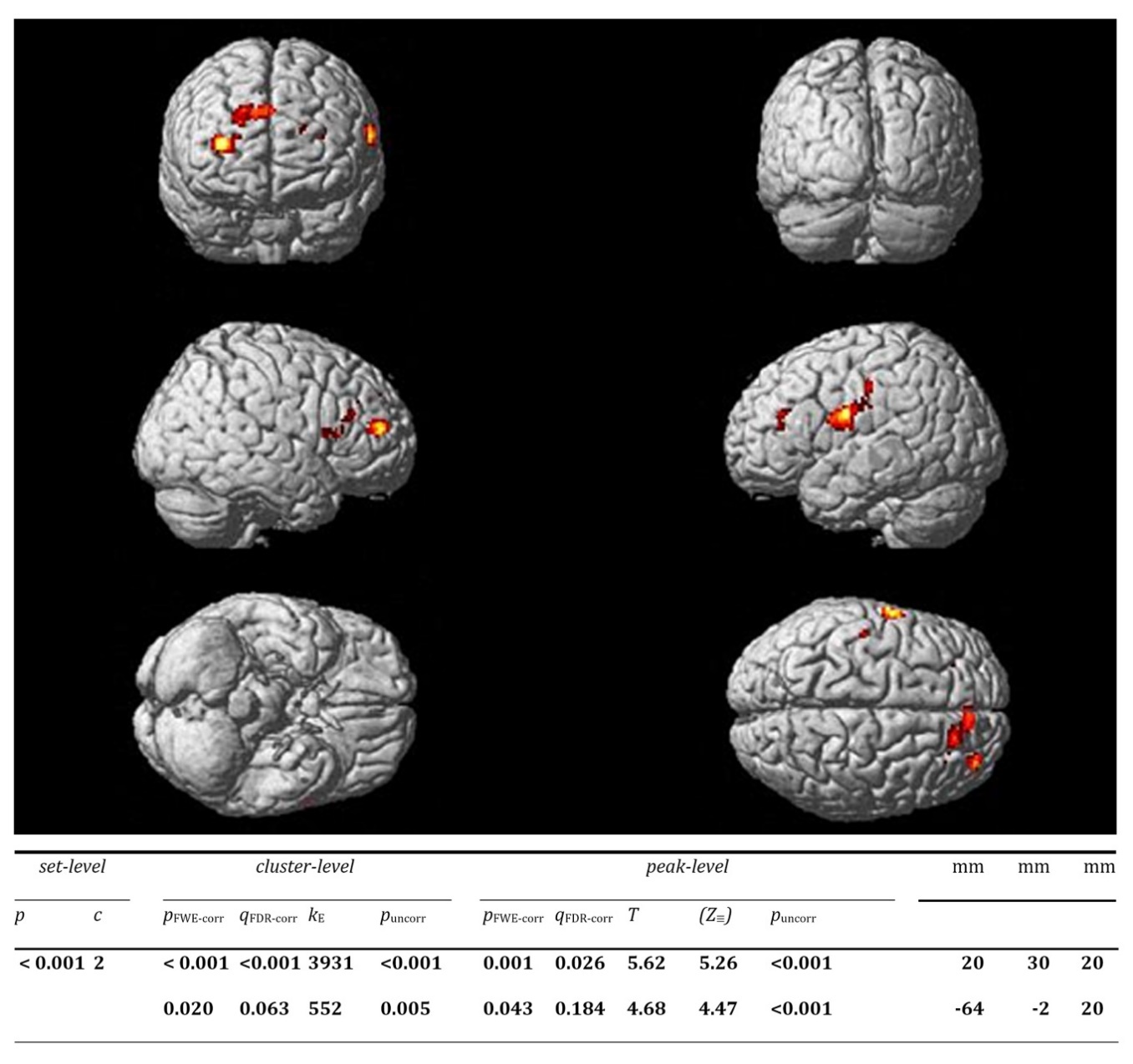

Supplement: Supplementary file 1 [file DataSheet_1.docx]
